# Supplementary figures and images for: Consortium-based genome-wide meta-analysis for childhood dental caries traits
Source: Hum Mol Genet. 2018 Jun 20;27(17):3113–27. doi: 10.1093/hmg/ddy237 (PMC6097157; doi:10.1093/hmg/ddy237)

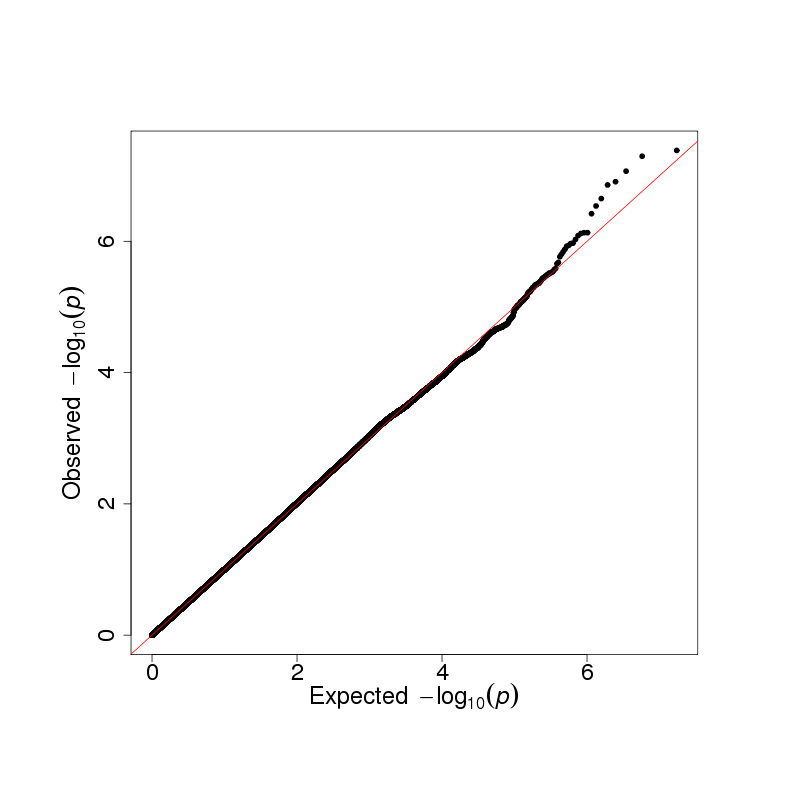

Supplement: Supplementary Figure [file ddy237_supp_sf_1.png]

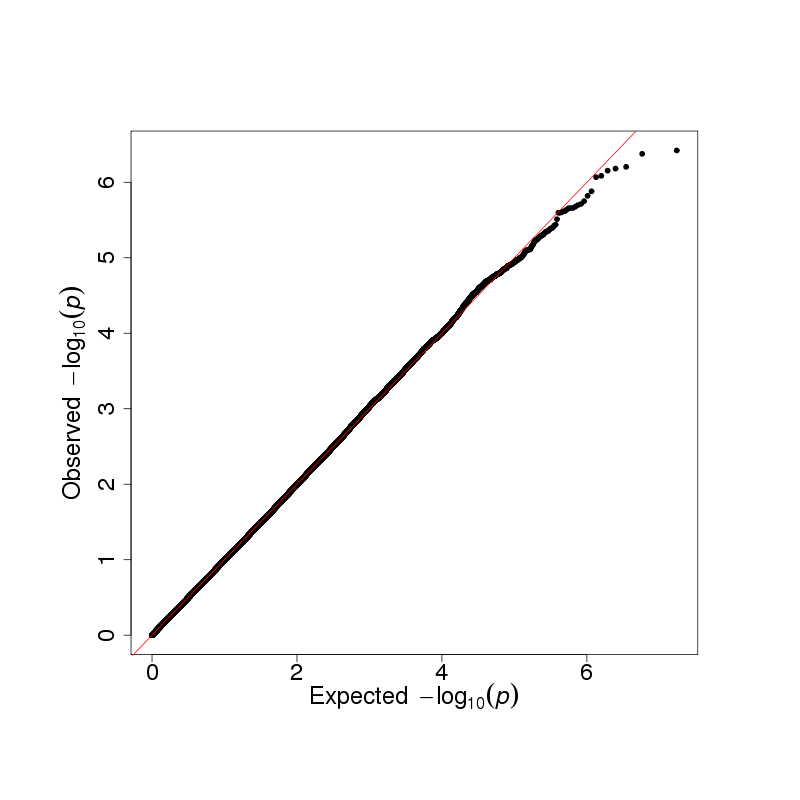

Supplement: Supplementary Figure [file ddy237_supp_sf_2.png]

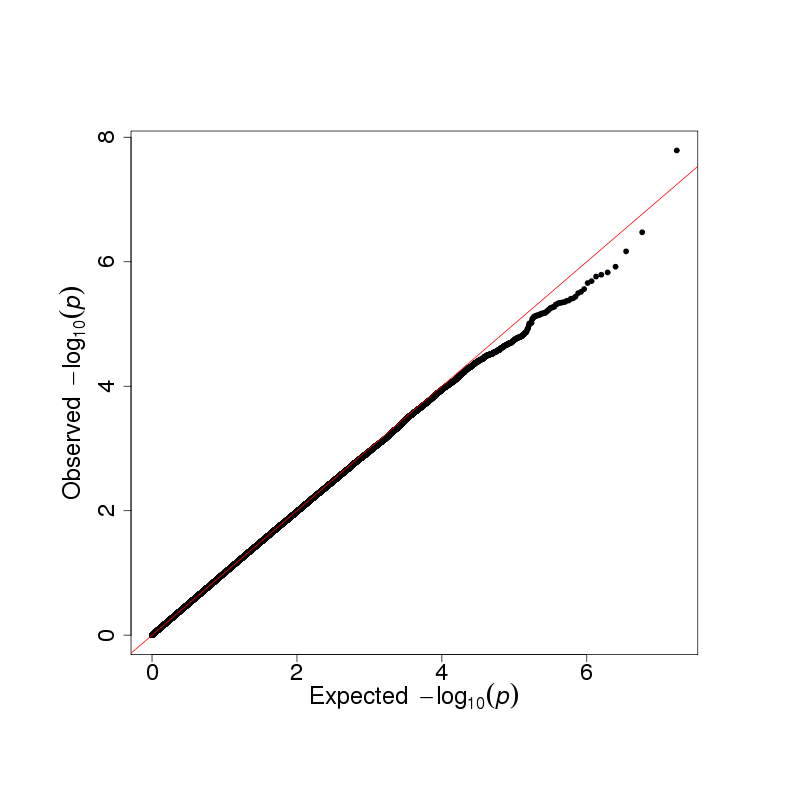

Supplement: Supplementary Figure [file ddy237_supp_sf_3.png]

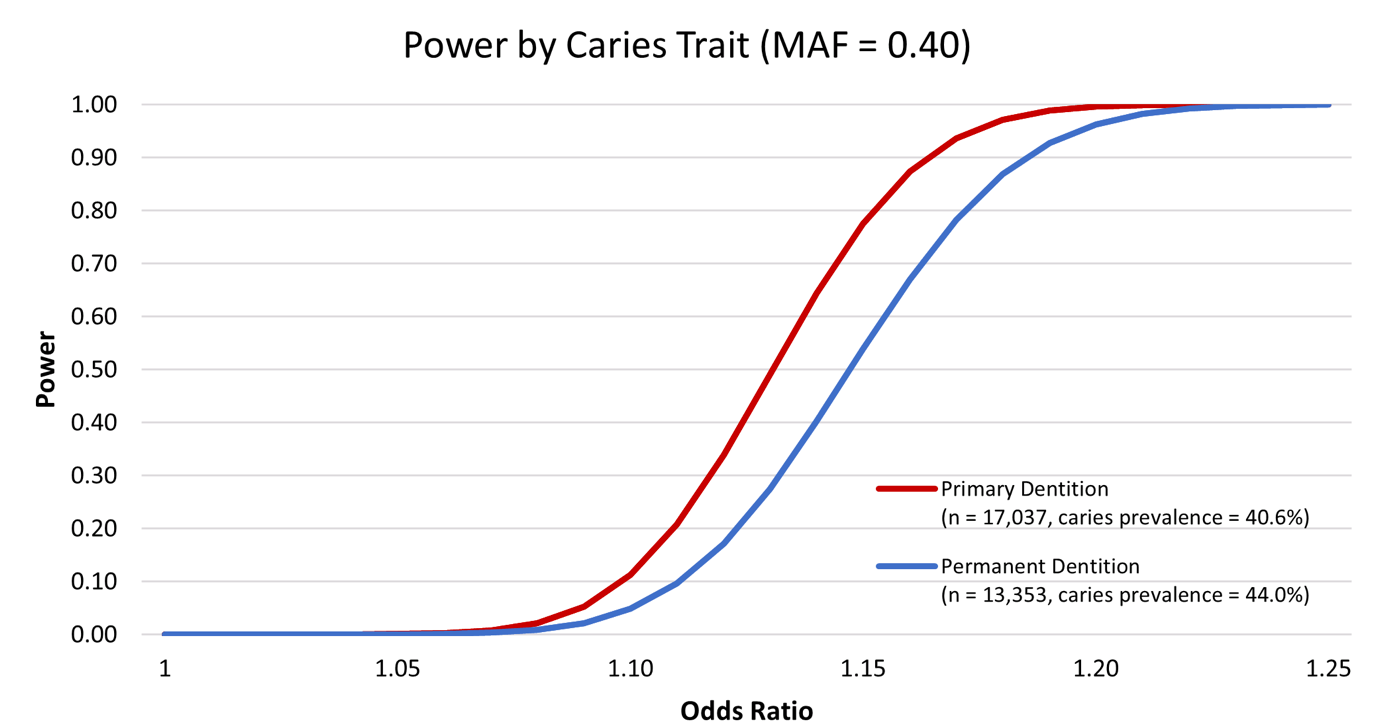

Supplement: Supplementary Figure [file ddy237_supp_sf_4.png]

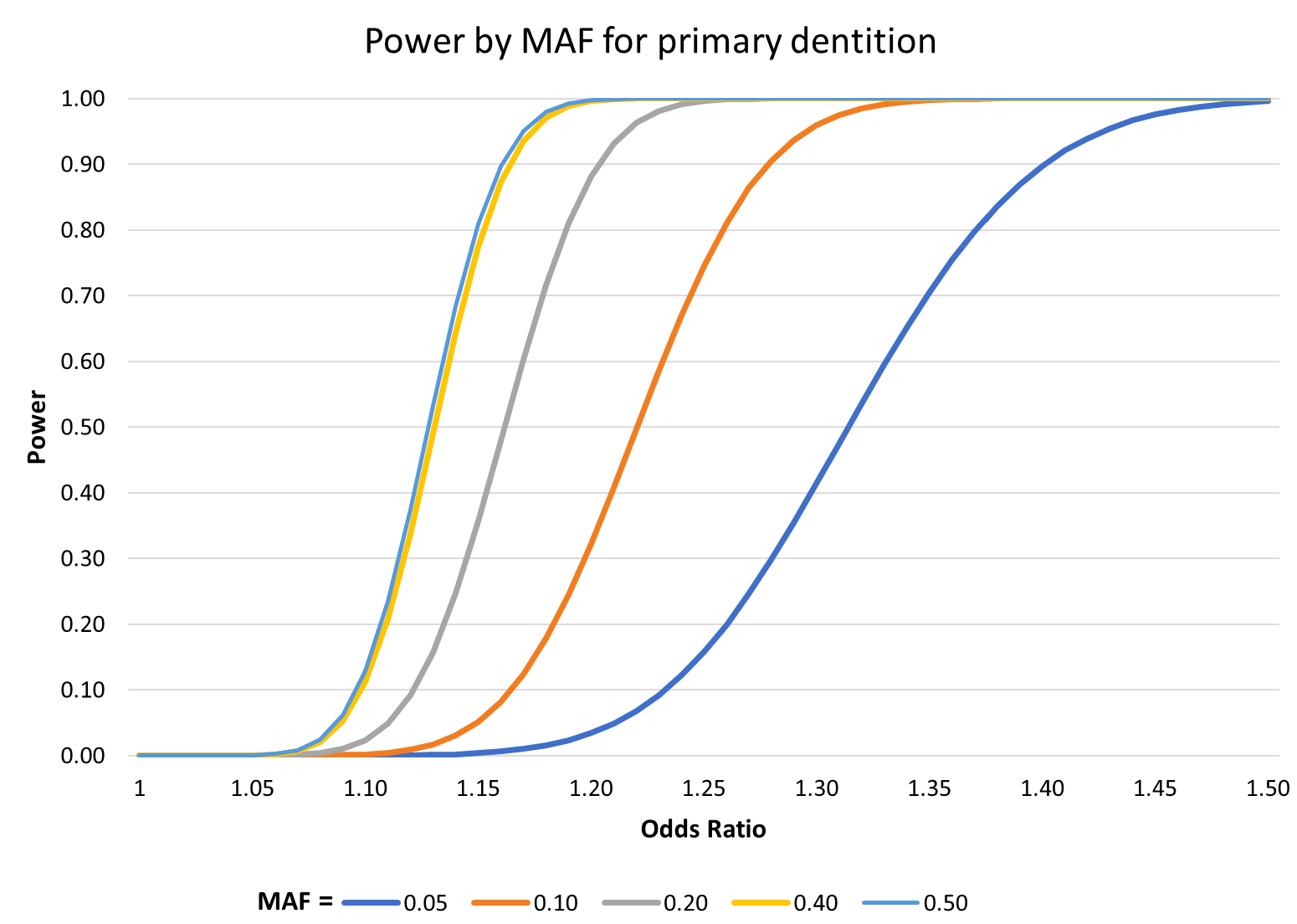

Supplement: Supplementary Figure [file ddy237_supp_sf_5a.png]

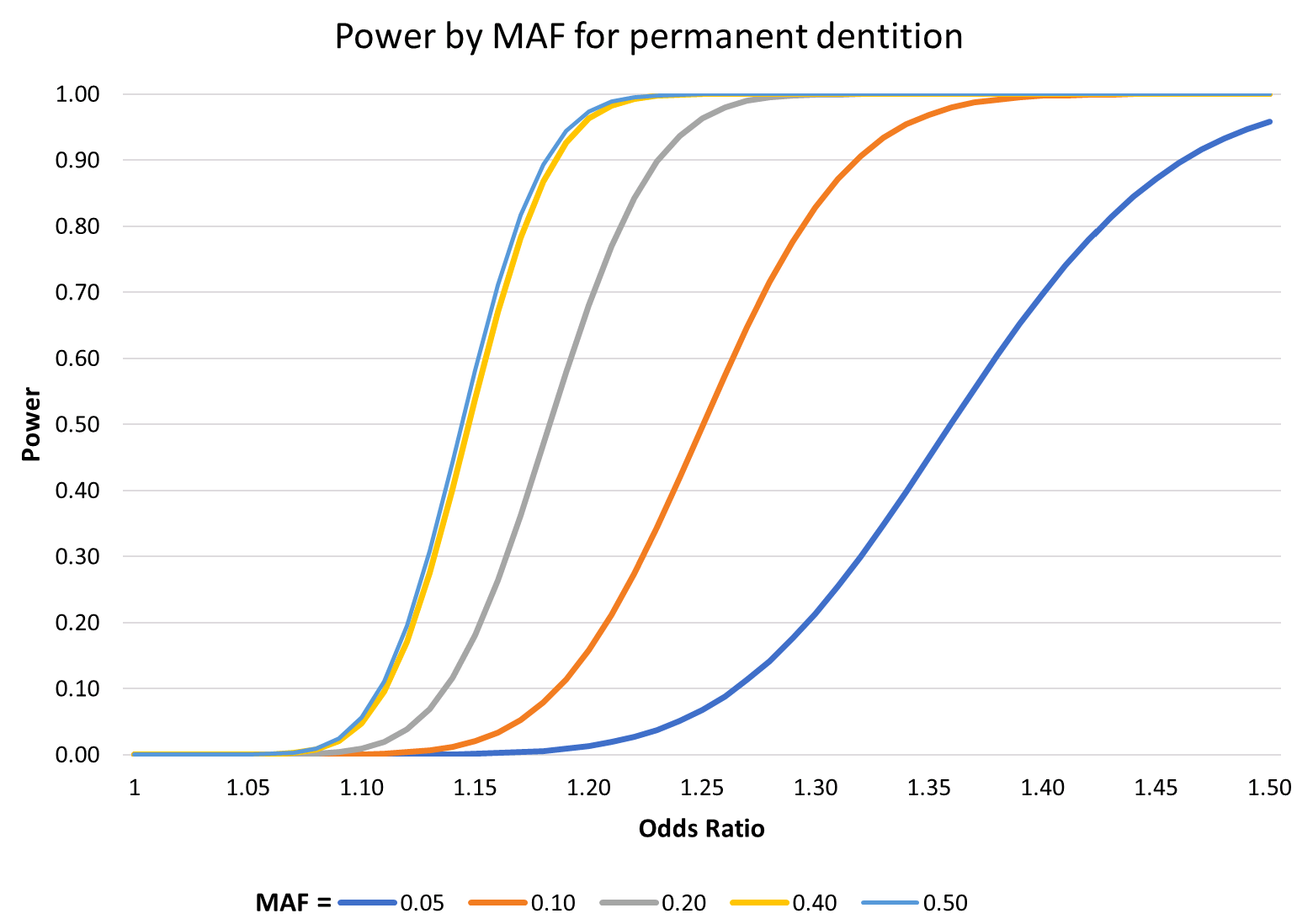

Supplement: Supplementary Figure [file ddy237_supp_sf_5b.png]
